# Bacterial composition of nasal discharge in children based on highly accurate 16S rRNA gene sequencing analysis

**DOI:** 10.1038/s41598-020-77271-z

**Published:** 2020-11-19

**Authors:** Kaoru Haro, Midori Ogawa, Mitsumasa Saito, Koichi Kusuhara, Kazumasa Fukuda

**Affiliations:** 1 Department of Microbiology, School of Medicine, University of Occupational and Environmental Health, Japan, 1-1 Iseigaoka, Yahatanishi-ku, Kitakyushu, 807-8555 Japan; 2 Department of Pediatrics, School of Medicine, University of Occupational and Environmental Health, Japan, Kitakyushu, Japan

**Keywords:** Microbiology, Diseases, Health care, Health occupations, Medical research

## Abstract

Nasopharyngeal colonization by bacteria is a prerequisite for progression to respiratory disease and an important source of horizontal spread within communities. We aimed to perform quantitative analysis of the bacterial cells and reveal the microbiota of the nasal discharge in children at the species level based on highly accurate 16S rRNA gene sequencing. This study enrolled 40 pediatric patients with rhinorrhea. The bacterial cells in the nasal discharge were counted by epifluorescence microscopic analysis. The microbiota was analyzed by using the 16S rRNA gene clone library sequencing method. We demonstrated that a high abundance (median 2.2 × 10^7^ cells/mL) of bacteria was contained in the nasal discharge of children. Of the 40 samples, 37 (92.5%) were dominated by OTUs corresponding to *Haemophilus aegyptius/influenzae*, *Moraxella catarrhalis/nonliquefaciens*, or *Streptococcus pneumoniae*. These samples showed higher cell abundance and lower alpha diversity than the remaining three samples in which the other bacteria coexisted. In addition, 12 sequences with low homology to type strains were considered as previously unknown bacterial lineages. In conclusion, the nasal discharge of most young children contains a large amount of respiratory pathogens and several unknown bacteria, which could not only cause endogenous infection but also be a source of transmission to others.

## Introduction

The nasopharynx of children contains respiratory pathogens, such as *Streptococcus pneumoniae*, *Haemophilus influenzae* and *Moraxella catarrhalis*^1^. Nasopharyngeal colonization with *S. pneumoniae* precedes the pathogenesis of pneumococcal diseases, such as acute otitis media, pneumonia, septicemia, and meningitis particularly among children^2–4^. In the elderly, *S. pneumoniae* is the most common cause of pneumonia and one of the most frequently isolated pathogens in patients with chronic obstructive pulmonary disease (COPD)^5^. *H. influenzae*, especially nontypeable *H. influenzae* (NTHi), is also a common bacterium associated with exacerbation of COPD and acute pneumonia^6,7^, and colonization of the upper respiratory tract is a prerequisite for developing respiratory disease^8,9^.

Carriage of these bacteria is also the primary mode of horizontal spread within communities^2^. Recent work has shown that young children play a major role in pneumococcal nasopharynx carriage, acquisition, and transmission^10^. Other previous studies have reported that respiratory viral infections increase nasopharyngeal pneumococcal transmission^11,12^. These findings suggest that nasal discharge can play an important role in the transmission of the bacteria colonizing the nasopharynx. In this regard, analysis of bacteria contained in the nasal discharge of children is important, since these bacteria may be transmitted among communities through nasal discharge and may occasionally cause infectious diseases depending on the condition of the host.

Recent studies conducted next-generation sequencing (NGS) have revealed age-related changes in the upper-airway microbiota^13^ and their association with respiratory tract infections^14,15^ and asthma^16,17^. However, details about the bacterial community at the species level in nasal discharge have not been well investigated. Therefore, we performed quantitative analysis of the bacterial cells and investigated the microbiota of the nasal discharge in children at the species level based on highly accurate 16S rRNA gene sequencing.

## Results

### Subject characteristics

The median age of the 40 enrolled children (22 males, 18 females) was 2.1 years (range, 0.2–7.0 years). The demographic and clinical characteristics of the patients are shown in Table 1.

**Table 1:** Demographic and clinical characteristics of the subjects.

Characteristics	
Age (year), median, (range)	2.1 (0.2–7.0)
Sex (male), n (%)	22 (55.0)
Antimicrobial drug use^a^, n (%)	13 (32.5)
Day care center^b^, n (%)	23 (67.6)
PCV7/PCV13 and Hib vaccinated (complete 4 doses)^c^, n (%)	24 (75.0)
**Symptom**	
Fever, n (%)	11 (27.5)
Cough, n (%)	36 (90.0)
Wheeze, n (%)	7 (17.5)
**Diagnosis**	
Acute upper respiratory tract inflammation, n (%)	22 (55.0)
Acute bronchitis, n (%)	8 (20.0)
Respiratory syncytial virus infection, n (%)	8 (20.0)
Allergic rhinitis, n (%)	1 (2.5)
Nasal congestion, n (%)	1 (2.5)

^a^Defined as use of antimicrobial drug within 1 month before sampling.

^b^No information available for 6 subjects.

^c^No information available for 8 subjects.

### Total bacterial numbers

The bacterial cells counted by epifluorescence microscopic analysis of the nasal discharge are shown in Supplementary Fig. 1. The numbers of bacteria of the samples ranged from 2.5 × 10^6^ to 3.2 × 10^8^ cells/mL (median 2.2 × 10^7^ cells/mL).

### Microbiota analysis

**Figure 1:**
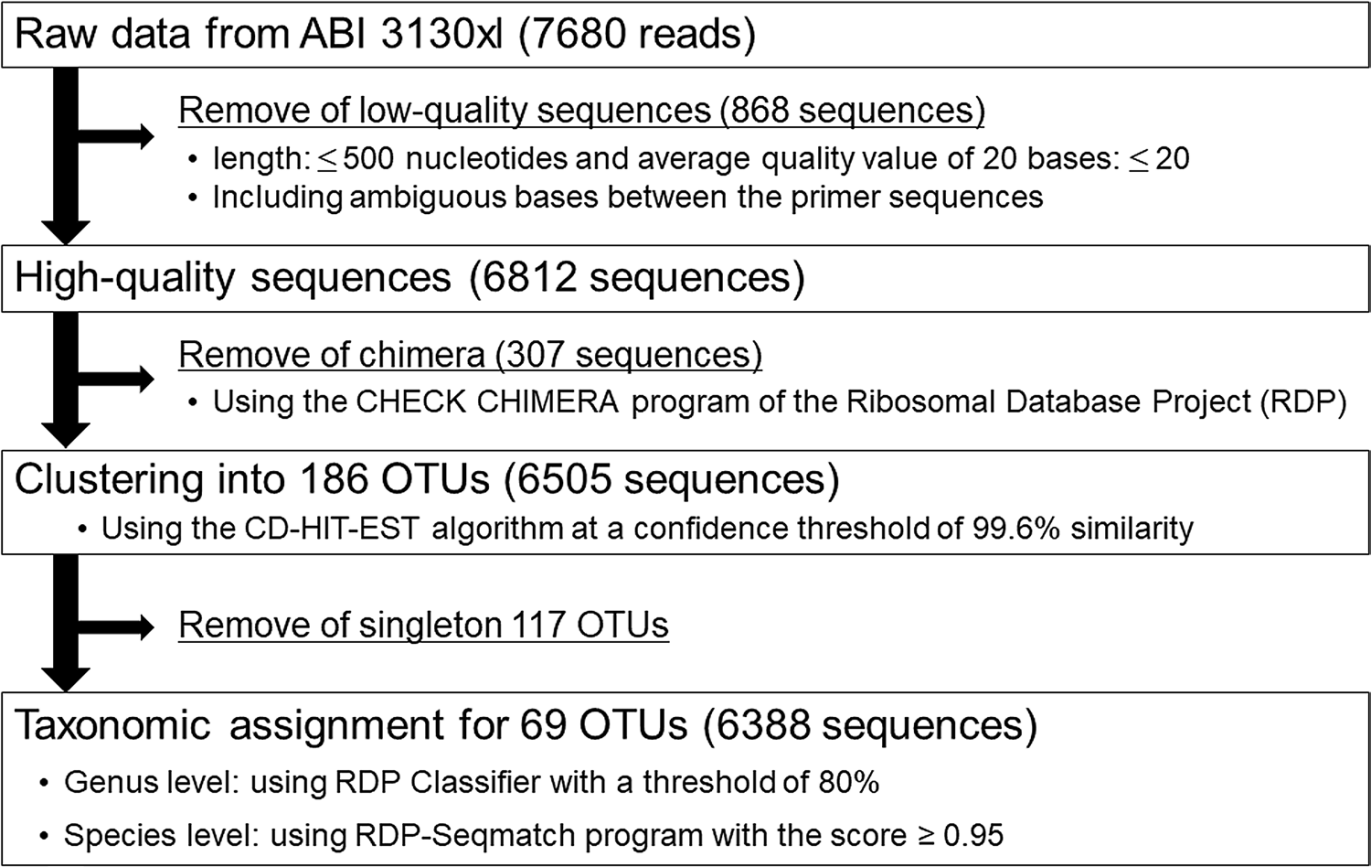
Flow chart of data analysis. *OTU* operational taxonomic unit.

We obtained 6812 high-quality sequences from 40 samples of nasal discharge (Fig. 1). Three hundred and seven chimeric sequences were removed, and 6505 sequences were clustered into 186 operational taxonomic units (OTUs), including 117 singletons, using a 99.6% sequence similarity threshold. Rarefaction curves for bacterial OTUs calculated for each sample and Good’s coverage are shown in Supplementary Fig. 2. The coverage including and excluding singletons ranged from 0.90 to 1.00 (median, 0.98) and from 0.95 to 1.00 (median, 0.99), respectively. The rarefaction curves were close to saturation in most of the samples with or without singletons. Therefore, singleton OTUs were removed from taxonomic assignment, and 69 OTUs remained. From the results of RDP-Classifier analysis, 66 of the 69 OTUs were identified at the genus level, of which 57 were identified at the species level based on a Seqmatch score.

**Figure 2:**
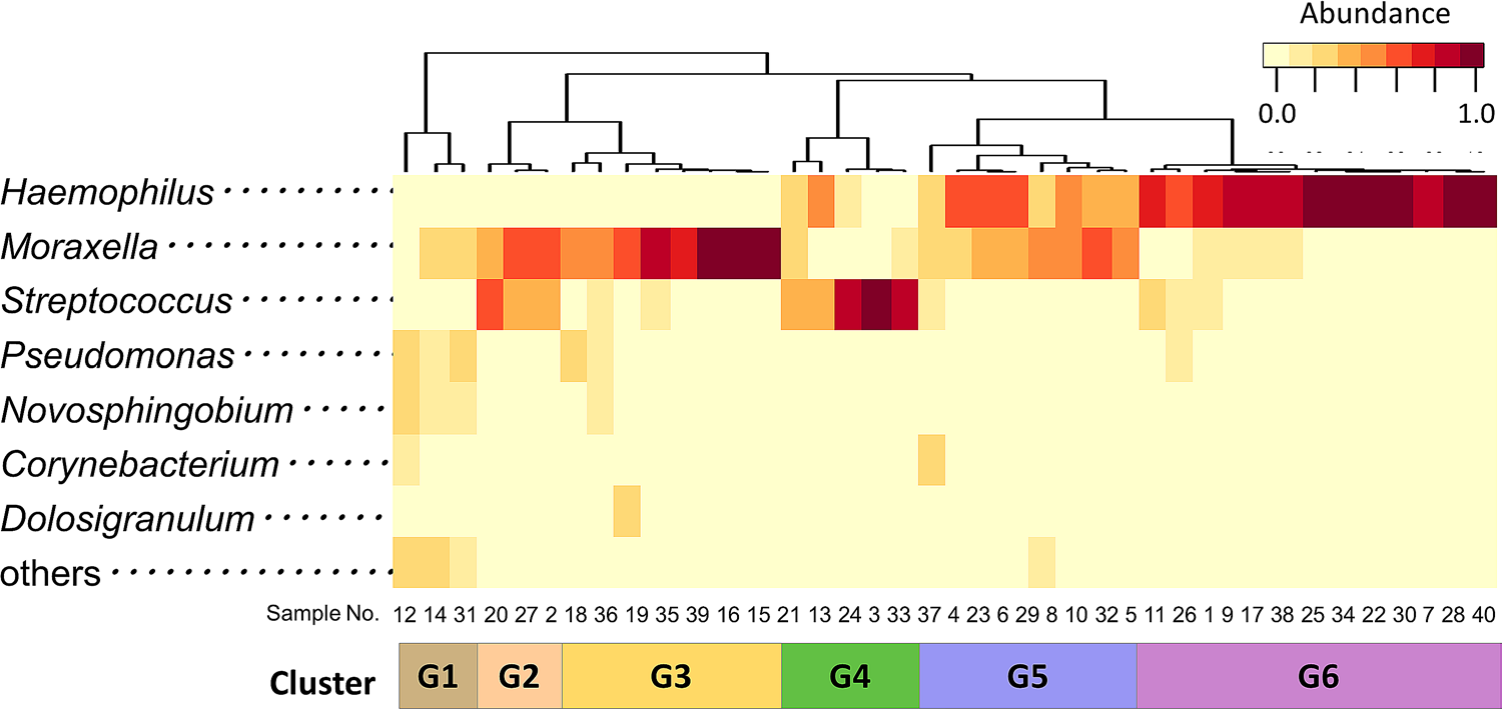
Clustering analysis of the OTUs at the genus level. Clustering of samples based on the relative abundance of the seven major genera and others (relative abundance < 1.0%) revealed six distinct clusters: G1, mixed; G2, *Moraxella* and *Streptococcus*-dominated; G3, *Moraxella*-dominated; G4, *Streptococcus*-dominated; G5, *Haemophilus* and *Moraxella*-dominated; G6, *Haemophilus*-dominated. Bacterial genera in the left row are arranged by frequency.

The relative abundance of all genera in each sample and the clustering results based on the composition of the genera are shown in Supplementary Fig. 3 and Fig. 2. The most abundant genera in nasal discharge were *Haemophilus* (40.1%), *Moraxella* (31.2%) and *Streptococcus* (15.4%), accounting for 86.7% of all sequences. Other fairly common genera were *Pseudomonas* (4.0%), *Novosphingobium* (3.0%), *Corynebacterium* (1.7%) and *Dolosigranulum* (1.0%). Clustering of samples based on the composition of the genera revealed six distinct clusters: G1) mixed, G2) *Moraxella* and *Streptococcus*- dominated, G3) *Moraxella*- dominated G4) *Streptococcus*- dominated, G5) *Haemophilus* and *Moraxella*- dominated, and G6) *Haemophilus*-dominated. Most of the samples (92.5%) were classified into clusters dominated by *Haemophilus*, *Moraxella*, *Streptococcus*, or a combination of these genera.

**Figure 3:**
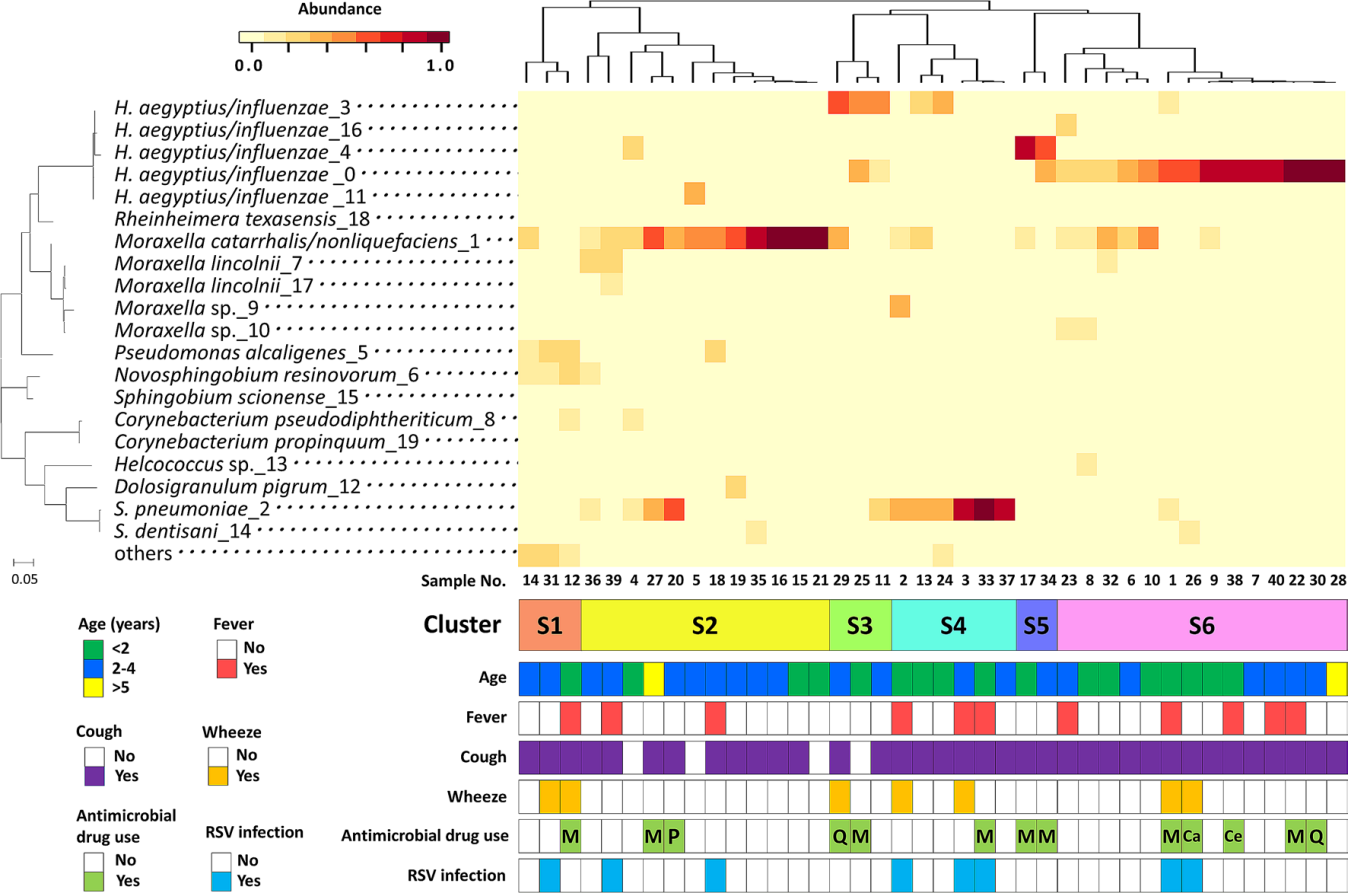
Clustering analysis of the OTUs at the species level and clinical information. The annotated bacterial species and the number of OTUs are shown on the left side of the heatmap. OTUs with relative abundance < 0.4% in the total sequences were grouped as “others”. Six distinct clusters were revealed by hierarchical clustering of samples based on the composition of the OTUs: S1, mixed; S2, *M. catarrhalis/nonliquefaciens* dominated; S3, *H. aegyptius/influenzae* (OTU 3) dominated; S4, *S. pneumoniae* dominated; S5, *H. aegyptius/influenzae* (OTU 4) dominated; S6, *H. aegyptius/influenzae* (OTU 0) dominated. Clinical information such as age, symptoms, antimicrobial drug use and diagnosis of respiratory syncytial virus infection are shown under each sample. *RSV* respiratory syncytial virus, *M* macrolide, *P* penicillin, *Q* new quinolone, *Ca* carbapenem, *Ce* cephem.

The relative abundance of all OTUs in each sample is shown in Supplementary Fig. 4. The clustering analysis of OTUs at the species level and clinical information for each sample are shown in Fig. 3. Six distinct clusters were revealed by hierarchical clustering of samples based on the composition of the OTUs: S1) mixed, S2) *M. catarrhalis/nonliquefaciens* dominated, S3) *H. aegyptius/influenzae* (OTU 3) dominated, S4) *S. pneumoniae* dominated, S5) *H. aegyptius/influenzae* (OTU 4) dominated and S6) *H. aegyptius/influenzae* (OTU 0) dominated. Thirty-seven (92.5%) of the 40 samples were classified into clusters dominated by OTUs corresponding to *H. aegyptius/influenzae*, *M. catarrhalis/nonliquefaciens*, or *S. pneumoniae*. The genera *Haemophilus*, *Moraxella* and *Streptococcus*, which were the three most abundant genera in the genus-level analysis, were composed of multiple OTUs in the species-level analysis. The genus *Haemophilus* was classified into 5 distinct OTUs representing *H. aegyptius/influenzae* (OTUs 0, 3, 4, 11 and 16) among the top 20 predominant OTUs. Sixteen samples had one of these types of OTUs, while 11 samples had two or more of these types of OTUs. Most of the OTUs classified into the genus *Moraxella* were classified into *M. catarrhalis/nonliquefaciens* in the species-level analysis. The others were classified into *M. lincolnii* or *Moraxella* sp. (unclassified at the species level). The genus *Streptococcus* was classified into two species: *S. pneumoniae and S. dentisani*.

**Figure 4:**
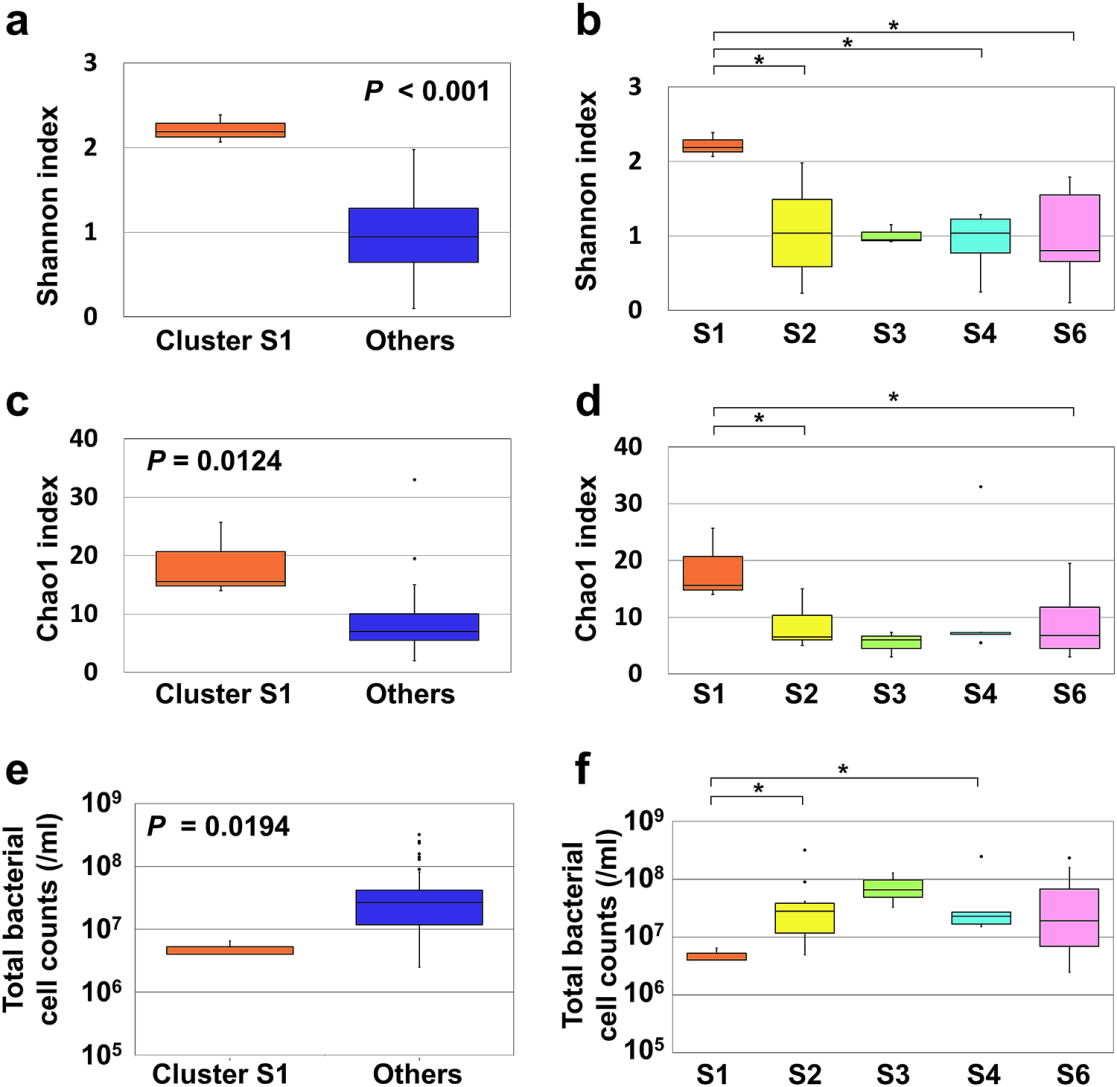
Comparison of the Shannon index, Chao1 index and bacterial cell counts between clusters based on the species-level analysis. The clusters were determined with clustering analysis at the species level (see Fig. 3). The Shannon index (**a**), Chao1 index (**c**) and total bacterial cell counts (**e**) of cluster S1 were compared with those of the other five clusters combined. The Shannon index (**b**), Chao1 index (**d**) and total bacterial cell counts (**f**) of cluster S1 were compared with those of the other four clusters. Cluster S5 was excluded from the analyses due to low subject numbers in cluster S5 (n = 2). The box plots show the 25th and 75th percentiles (bottom and top edges of the boxes, respectively), the median (middle horizontal line), and the smallest and largest values that are not outliers (top and bottom whiskers), with outlier defined as 1.5 times the interquartile range (points). Statistical significance between cluster S1 and the aggregate of the five clusters was tested using the Mann–Whitney U test. Comparing cluster S1 to each four clusters, the Steel test was applied (bars; *p < 0.05).

Comparisons of the bacterial cell counts and alpha diversity (Shannon and Chao1 indices) between clusters are shown in Fig. 4. The Shannon index was significantly higher in Cluster S1 (mixed) than in the aggregate of the other five clusters (dominated by *H. aegyptius/influenzae*, *M. catarrhalis/nonliquefaciens*, or *S. pneumoniae*) (Fig. 4a, p < 0.001) or in cluster S2, cluster S4 and cluster S6 (Fig. 4b). The Chao1 index was significantly higher in Cluster S1 than in the aggregate of the five clusters (Fig. 4c, p = 0.0124) or in cluster S2, and cluster S6 (Fig. 4d). In contrast, cluster S2, cluster S4 and the aggregate of the five clusters had significantly higher bacterial cell counts than cluster S1 (Fig. 4e,f). The clinical and demographic characteristics of cluster S1 showed no significant differences compared to those of any other cluster except for wheezing in cluster S1 versus cluster S2 (Table 2).

**Table 2:** Comparison of clinical and demographic characteristics between clusters based on the species-level analysis.

Cluster	S1 (n = 3)	S2 (n = 12)	S3 (n = 3)	S4 (n = 6)	S5 (n = 2)	S6 (n = 14)
Age (year), median	2.3	2.1	2.0	1.9	2.0	1.9
Sex (male), n (%)	1 (33)	6 (50)	1 (33)	5 (83)	1 (50)	8 (57)
Fever, n (%)	1 (33)	2 (17)	0 (0)	3 (50)	0 (0)	5 (36)
Cough, n (%)	3 (100)	9 (75)	2 (67)	6 (100)	2 (100)	14 (100)
Wheeze, n (%)	2 (67)	0 (0)*	1 (33)	2 (33)	0 (0)	2 (14)
Antimicrobial drug use^a^	1 (33)	2 (17)	2 (67)	1 (33)	2 (100)	5 (36)
Day care center^b^, n (%)	1 (50)	4 (40)	2 (100)	4 (67)	2 (100)	10 (83)
Hib vaccination completed^c^, n (%)	3 (100)	7 (70)	1 (100)	5 (83)	2 (100)	6 (60)
PCV vaccination completed^c^, n (%)	3 (100)	7 (70)	1 (100)	5 (83)	2 (100)	6 (60)
RSV infection, n (%)	1 (33)	2 (17)	0 (0)	3 (50)	0 (0)	2 (14)

*RSV* Respiratory syncytial virus, *Hib* Haemophilus influenzae type b, *PCV* pneumococcal conjugate vaccine.

^a^Defined as use of antimicrobial drug within 1 month before sampling ^b^No information available for 6 samples.

^c^No information available for 8 samples.

*p < 0.05 compared with cluster S1 by Fisher's exact test.

### Unclassified clusters

Nine OTUs of all 69 OTUs analyzed in this study were unclassified at the species level based on a Seqmatch score ≥ 0.95 (Table 3). Two OTUs (OTU 42, OTU 59) were unclassified at the genus level but were identified at the family level, whereas 1 OTU (OTU 30) was unclassified at even the phylum level based on assignment using the RDP Classifier with a confidence threshold of 0.8. The analysis using the RDP Classifier showed that the representative sequences of OTUs 42 and 59 showed 75% and 77% similarity, respectively, with the genus *Snodgrassella*, and 100% similarity with the family *Neisseriaceae*. According to the results, OTUs 42 and 59 were possibly previously unknown bacteria at the genus level within the family *Neisseriaceae*. NCBI BLAST search (https://blast.ncbi.nlm.nih.gov) results revealed that the representative sequence of OTU 30 shared 99.8% similarity with an unclassified bacterium. Recently, the unclassified bacterium was identified in bronchoalveolar lavage fluids (BALFs) in adults with respiratory disease and was temporally named IOLA (Infectious Organism Lurking in Airways)^18^. IOLA was also detected in specimens from patients with respiratory tract disease and in pig lungs^19–21^. To detect IOLA, a nested PCR performed on all 40 samples revealed that 4 samples (10%) were positive for IOLA (Supplementary Fig. 5).

**Table 3:** Unclassified OTUs (possibly previously unknown bacteria).

Phylum level (less than 80% RDP classifier score to known bacterial type strains)				
OTU No	Domain (RDP-classifier score)	Phylum (RDP-classifier score)	Number of samples	Number of clones
30	Bacteria (96%)	*Bacteroidetes* (61%)	1	8

Genus level (less than 80% RDP-classifier score to known bacterial type strains)				
OTU No	Family (RDP-classifier score)	Genus (RDP classifier score)	Number of samples	Number of clones
42	*Neisseriaceae* (100%)	*Snodgrassella* (75%)	1	3
59	*Neisseriaceae* (100%)	*Snodgrassella* (77%)	1	2

Species level (less than 0.95 Seqmatch score to known bacterial type strains)				
OTU No	Genus (RDP-classifier score)	Species (Seqmatch score)	Number of samples	Number of clones
9	*Moraxella* sp. (99%)	*M. lincolnii* (0.868)	4	72
10	*Moraxella* sp. (100%)	*M. lincolnii* (0.940)	5	69
13	*Helcococcus* sp. (100%)	*Helcococcus ovis* (0.818)	7	53
27	*Flavobacterium* sp. (100%)	*Flavobacterium suzhouense* (0.825)	4	12
28	*Rheinheimera* sp. (100%)	*Rheinheimera aquatica* (0.924)	6	10
29	*Haemophilus* sp. (100%)	*H. aegyptius* (0.942)	1	8
37	*Haemophilus* sp. (89%)	*Actinobacillus minor* (0.885)	2	4
41	*Pseudomonas* sp. (99%)	*Pseudomonas alcaligenes* (0.921)	2	3
43	*Haemophilus* sp. (91%)	*H. sputorum* (0.895)	2	3

Phylogenetic trees based on 16S rRNA gene sequences, including unclassified clones in this study and type strains belonging to the same genus or family, are shown in Supplementary Figs. 6–12. The results of the phylogenetic analysis showed that the unclassified clones in this study were located in a separate branch from previously known type strains.

## Discussion

In this study, we found a high abundance (median 2.2 × 10^7^ cells/mL) of bacteria in the nasal discharge of children, and most of the samples were dominated by OTUs corresponding to *H. aegyptius/influenzae*, *M. catarrhalis/nonliquefaciens*, or *S. pneumoniae*, which are potentially pathogenic bacteria associated with respiratory diseases in both children and adults^22,23^.

In addition, the samples dominated by these pathogenic bacteria showed higher cell abundance and lower alpha diversity than those in which the other bacteria coexisted. These results suggest that most young children have a large amount of pathogens in their nasal discharge, which may be not only a cause of infections in those children but also a source of pathogens that can be transmitted to others. Our results are consistent with previous studies on the nasopharyngeal microbiota in children, that revealed a relatively high abundance of the genera *Moraxella*, *Streptococcus*, *Haemophilus*, *Staphylococcus, Corynebacterium* and *Dolosigranulum*^24^. However, most of these studies used nasal swabs for specimen collection and performed no quantitative evaluation of bacteria in nasal discharge. Furthermore, these previous studies on the nasal microbiota were analyzed by NGS. Although NGS has been widely applied in human microbiota studies, NGS analyses are often insufficient at the species level because respiratory samples contain many kinds of *Streptococcus* spp. which show high similarity in their 16S rRNA gene sequences^25^. In our analysis, the sequences determined by the Sanger method were clustered into OTUs at a threshold of 99.6% sequence similarity, indicating that sequences with more than two base differences were clustered into different OTUs. These methods make it possible to accurately identify the bacterial flora in nasal discharge at the species level.

Our findings are consistent with a previous report that indicated that the genera *Moraxella*, *Streptococcus* and *Haemophilus* colonized the nasopharynx of children significantly more frequently in acute respiratory infection than in noninfection states^14^. Many of our study subjects who visited a pediatric clinic with rhinorrhea did not have fever (72.5% nonfever). Such children often participate in normal life activities, such as attending a daycare center. However, from the viewpoint of transmission of pathogenic bacteria within a community, we consider that even rhinorrhea, which is considered a mild symptom, plays an important role as a source of infection. Transmission of nasopharyngeal bacteria has been most commonly reported for *S. pneumoniae*^2^. Mosser et al. showed that children play an important role in bringing pneumococci into and maintaining carriage and transmission within households^10^. In this study, not only *S. pneumoniae* but also several bacteria related to respiratory diseases in adults, such as the genara *Haemophilus*, *Moraxella*, and *Novosphingobium*, were observed in the nasal discharge of children. *H. influenzae* is a cause of community acquired pneumonia in adults as well as children^26^, and the most common cause of exacerbation of COPD^27^. *Moraxella* and *Novosphingobium* have also been reported to associate with COPD^28,29^. Colonization by these bacteria in the upper respiratory tract is a prerequisite for the development of invasive infections^2,3,8,9^. Therefore, our results indicate that contact with children with rhinorrhea in closed communities (families, children attending schools or daycare centers etc.) may be a risk factor for community-acquired pneumonia and COPD exacerbation.

In our analysis, there were five different OTUs with the same taxonomy, namely, “*H. aegyptius/influenzae*”, among the top 20 predominant OTUs (OTUs 0, 3, 4, 11 and 16). Several samples had two or more of these OTUs. Simultaneous colonization with several different *H. influenzae* strains is often observed^30,31^. Therefore, our results indicate that there were multiple strains of *H. influenzae* in the nasal discharge of children. In addition, with the widespread use of Hib vaccination, the incidence of invasive infection caused by NTHi has recently increased^32^. Several studies also reported nosocomial outbreaks of NTHi infection^33,34^. Although analysis targeting the 16S rRNA gene sequence cannot identify serotypes among strains, pathogenicity, and antibacterial susceptibility, high-precision analysis based on this gene would be useful for understanding disease transmission and tracking the spread of strains^35^.

In this study, using the Sanger method targeting 550 bp of the 16S rRNA gene including three variable regions (V3, V4 and V5), most of the sequences were classified into known species and only twelve OTUs (composed of 239 sequences) of 69 OTUs (6388 sequences) were assigned to unclassified bacteria at the species level or higher. One of the twelve OTUs unclassified at the phylum level using the RDP Classifier showed high similarity with IOLA according to the NCBI BLAST search. We have previously reported that IOLA is a novel microorganism found in BALFs in adults with respiratory disease^18^. This is the first report of IOLA detected in nasal discharge of children. In this study, IOLA was detected by nested-PCR in four samples (10%), which suggested that children are colonized or infected with IOLA in the nasal cavity at a certain rate. IOLA is unlikely to have an obvious influence on the health of children but might have some effects because IOLA has been detected in the human respiratory tract only in patients with lung disease^18–20^. In this study, we also detected some sequences assigned as previously unknown species in the genera *Moraxella, Helcococcus, Flavobacterium, Rheinheimera, Haemophilus,* and *Pseudomonas* and in the family *Neisseriaceae*. Thus, many previously unknown bacteria may be contained in nasal discharge. Further investigation is needed to clarify the influence of these bacteria on human health and disease.

This study has some limitations. First, our small sample size may have been insufficient to detect associations between nasal discharge microbiota patterns and the clinical and demographic characteristics of patients. Further studies are needed to clarify the role of the microbiota of nasal discharge in development of disease and symptom expression, and to evaluate the influence of antimicrobial drug use, vaccination, and environmental factors on the microbiota. Second, the samples for DNA analysis were stored at 4ºC until time of analysis (within 7 days: average 2.4 days). In preliminary experiments, we confirmed that major bacterial constitutions in a sample were not significantly changed during store at 4 °C for 5 days by using a clone library method. However, the possibility cannot be denied that storage at 4 °C may have some effect on the bacterial composition. Finally, the number of clones identified in this study was a maximum of 192 per sample, suggesting that this method cannot evaluate bacterial species in minor populations (approximately < 0.5%). In fact, IOLA was detected in only one sample by the clone library but was detected in four samples by specific PCR in this study. However, the rarefaction analysis in this study revealed that the clone library was sufficient to evaluate the bacterial diversity of nasal discharge in the major population.

In conclusion, we found that the potentially pathogenic bacteria associated with respiratory disease were predominantly detected at high abundance in most samples of nasal discharge from children. Furthermore, several previously unknown bacteria were discovered by highly accurate 16S rRNA gene sequencing. Close contact with children with rhinorrhea may be a risk factor for developing or exacerbating respiratory diseases especially for the elderly and patients with underlying diseases.

## Methods

### Subject enrollment

Forty patients who visited the Sato Children’s Clinic, Kitakyushu, Japan, with rhinorrhea in January 2017 were enrolled in this study. Clinical and demographic information was collected using a standardized questionnaire. The questionnaire completed by nurses or physicians at the time of enrollment contained information about gender, date of birth, symptoms, clinical diagnosis, and antimicrobial drug use within 1 month before sampling. Rapid antigen detection tests for adenovirus (ImunoAce Adeno, TAUNS Laboratories Inc., Shizuoka, Japan), influenza virus (ImunoAce Flu, TAUNS Laboratories Inc.), and respiratory syncytial virus (ImunoAce RSV Neo, TAUNS Laboratories Inc.) were performed when clinically indicated. Additional clinical information about daycare centers and vaccination status with a 7- valent or, 13-valent pneumococcal conjugate vaccine (PCV) and *H. influenzae* type b (Hib) vaccine was collected using a chart review.

### Ethics statement

This study was approved by Ethics Committee of Medical Research, University of Occupational and Environmental Health, Japan (No. H28-184). All experiments and methods in this study were performed in accordance with relevant guidelines and regulations. Informed consent was obtained from all parents/legally authorized representatives as children are involved in the study.

### Sample collection

The nasal discharge was aspirated through a nasal olive tip (AS ONE Corporation, Osaka, Japan) attached to a Specimen Collection Container (Nippon Covidien Ltd., Tokyo, Japan) from both nostrils by a trained nurse. The samples were stored immediately at 4 °C until analysis (within 7 days: average 2.4 days). Each nasal discharge sample was diluted 10 times with phosphate-buffered saline (PBS) and vigorously shaken by a Micro Smash MS-100 apparatus (Tomy Seiko Co., Ltd., Tokyo, Japan) for 15 s at 4,500 rpm. The diluted sample was used for cell counting and DNA extraction.

### Epifluorescence staining method

The total bacterial cells in the sample were counted by a epifluorescence staining method^36^. One-hundred micro-liter of the diluted nasal discharge sample was added to 900 μL of 4′,6-diamidino-2-phenylindole (DAPI) solution (1 μg/mL in 0.1 M phosphate [pH 8.5] buffer, 5% NaCl, 0.5 mM sodium EDTA) and then, incubated at room temperature for 20 min. After washing with 2 mL of water filtrated with a filter pore-size 0.2 μm, objects shaped like bacteria were counted under an Olympus BX40 epifluorescence microscope (Olympus Optical, Tokyo, Japan), and the number of bacteria per milliliter of nasal discharge specimen was then calculated^37^. All samples were counted 3 times by the author and coauthor.

### DNA extraction method

A 630-μL aliquot of the nasal discharge diluted 10 times with PBS as mentioned above was mixed with 70 μL of 30% sodium dodecyl sulfate (final concentration, 3.0%) and approximately 0.3 g of a mixture of glass beads that consisted of equal weights of 0.1 mm- and 1 mm-diameter beads. The mixture was vigorously shaken by a Micro Smash MS-100 apparatus (Tomy Seiko Co., Ltd.) for 30 s at 5500 rpm. Then, the mixture was treated with 500 μL of TE-saturated phenol (Nacalai Tesque, Kyoto, Japan) (vortex-mixed for 30 s) and centrifuged at 15,000 rpm for 5 min. DNA in the aqueous phase was washed with PBS buffer and TE buffer (10 mM Tris–HCl, 1 mM EDTA-2Na, [pH8.0]) two times and concentrated to a final volume of approximately 50 μL in TE buffer using Amicon Ultra-100K filters (Merck Millipore Ltd., Billerica, MA, USA). DNA extracted from 630-μL of PBS without nasal discharge was used as negative control template.

### Polymerase chain reaction conditions

The 16S rRNA genes were amplified as described previously^37^. Briefly, 25- µL PCR mixtures containing the universal primer set^38^ (E341F; 5′-CCTACGGGAGGCAGCAG-3′ and E907R; 5′-CCGTCAATTCMTTTRAGTTT-3′) and AmpliTaq Gold DNA Polymerase LD (Applied Biosystems, Foster City, CA) were prepared. Then the reaction mixtures were incubated at 96 °C for 5 min, followed by 30 cycles of 96 °C for 30 s, 53 °C for 30 s and 72 °C for 1 min and a final elongation step of 72 °C for 7 min. The PCR products were evaluated by 2% agarose gel electrophoresis. No DNA band was observed in the negative control samples.

### Clone library construction and nucleotide sequencing analysis

All PCR products except for a negative control were confirmed as positive using 2% agarose gel electrophoresis and cloned using the TOPO TA Cloning Kit (Invitrogen, Carlsbad, California). A total of 192 colonies from each sample were randomly selected for sequencing analysis. Sequencing analysis was performed as described previously^36^. Briefly, the partial fragments of the cloning vectors (pCR II) containing inserted PCR products were amplified with AmpliTaq Gold 360 DNA Polymerase and a primer set (M13Forward: 5′-GTAAAACGACGGCCAG-3′ and M13Reverse: 5′-CAGGAAACAGCTATGAC-3′). After the primers and deoxyribonucleotide triphosphate were eliminated from the PCR mixture with an ExoSAP-IT (GE Health care UK Ltd., England, UK) according to the manufacturer’s instructions, an aliquot (1 µL) was used for the sequencing reaction. The sequencing reactions were accomplished with the primer ‘‘M13Forward’’ and the BigDye Terminator Cycle Sequencing Kit v3.1 (Applied Biosystems). The nucleic acid sequences were determined on a 3130xl Genetic Analyzer (Applied Biosystems).

Data analysis was performed using ‘Sequencing Analysis V.5.2′ software (Applied Biosystems) as described previously^39^. The sequences remaining after the quality check (read length: > 500 nucleotides and average quality value of 20 bases: ≥ 20) and only the reads containing precise sequences of the universal primers E341F and E907R were selected. In addition, the raw data were checked, and sequences including ambiguous bases between the primer sequences were excluded. The remaining highly accurate sequences were trimmed to remove primer and vector sequences. Approximately 550 base pairs were screened for chimeras with the Check Chimera program of the Ribosomal Database Project (RDP)^40^. Then, the sequences were clustered into OTUs^41^ using the cd-hit-est algorithm^42^ at a threshold of 99.6% sequence similarity. Representative OTU sequences were classified at the genus using the RDP Classifier^43^ with a confidence threshold of 80%. Sequences with less than 80% were deemed unclassified and classified into the next higher taxonomic rank with RDP Classifier confidences greater than 80%. Species level taxonomies were also assigned by using the RDP-Seqmatch program according to the highest score. When no species matched the sequence with a Seqmatch score ≥ 0.95, the sequences were deemed unclassified at the species level. *Haemophilus aegyptius* and *H. influenzae* were described as “*H. aegyptius/influenzae*” because they cannot be distinguished by their 16S rRNA genes^44^. In the same way, *M. catarrhalis* and *M. nonliquefaciens* were described as “*Moraxella catarrhalis/nonliquefaciens*” because they cannot be distinguished by analysis of the 550 bp of the 16S rRNA gene targeted in this study. For the species‐level analysis, different OTUs with the same taxonomy were distinguished by OTU number following the taxonomy name.

### Detection of IOLA

Nested PCR was performed on all samples for detection of IOLA. The 16S rRNA genes were amplified with a universal primer set as mentioned above (25 cycles). Subsequent PCR was performed using IOLA specific primers (30 cycles) as described previously^18^.

### Data analysis

All data analyses were performed using R software V3.6.1. Heatmaps were generated using the heatmap3 package. To assess the diversity coverage of the clone library, rarefaction curves were obtained using the vegan package. Coverage was calculated according to the equation proposed by Good^45^, where C = 1 − (n / N), n = the number of OTUs represented by only one clone and N = the total number of clones. Alpha-diversity assessed by the Shannon and Chao1 indices was calculated using the estimateR function in the vegan package.

### Phylogenetic analysis

In all phylogenetic analyses to classify the twelve unclassified sequences in this study, the data sets of nucleic acid sequences were obtained from the RDP databases. The multiple alignments were built using MAFFT^46^ with default settings. IQ-TREE software v1.6.10^47^ was used to construct maximum likelihood (ML) trees, and the appropriate models for each phylogenetic analysis were selected by Model Finder. The trees were visualized with FigTree v1.4.4. All sequences were submitted to a public database (DNA Data Bank of Japan, accession number: LC558357-LC564861).

### Statistical analysis

Comparisons of the proportions of sex, symptoms and antimicrobial drug use were analyzed by Fisher's exact test. The Kruskal–Wallis test and Mann–Whitney U test were used to compare the median age, the Shannon index, the Chao1 index and the total bacterial counts among groups of clusters. The Steel test was used to compare cluster S1 to each four clusters. P-values less than 0.05 were considered to be statistically significant. STATA 14.2 software (Stata Corporation, College Station, TX), BellCurve for Excel, version 3.20 software (Social Survey Research Information, Tokyo, Japan), and R software V3.6.1. were used for the analysis.

## Supplementary information

Supplementary Information.
