# Supplementary material for: Bacterial composition of nasal discharge in children based on highly accurate 16S rRNA gene sequencing analysis
Source: Sci Rep. 2020 Nov 19;10:20193. doi: 10.1038/s41598-020-77271-z (PMC7678852; doi:10.1038/s41598-020-77271-z)
Supplement: Supplementary file 1 — Supplementary Information. [file 41598_2020_77271_MOESM1_ESM.pdf]

Bacterial composition of nasal discharge in children based on highly accurate 16S rRNA gene sequencing analysis

Authors: Kaoru Haro[1,2], MD, Midori Ogawa[1], PhD, Mitsumasa Saito[1], MD, PhD, Koichi Kusuhara[2], MD, PhD, Kazumasa Fukuda[1], PhD

Affiliations: [1]Department of Microbiology, School of Medicine, University of Occupational and Environmental Health, Japan, Kitakyushu, Japan, [2] Department of Pediatrics, School of Medicine, University of Occupational and Environmental Health, Japan, Kitakyushu, Japan

Supplementary Figure Legends

Supplementary Figure 1: Total bacterial cell counts in each sample.

The samples are ordered by clusters based on species-level analysis (see Figure 3). The medians of the total bacterial cell count per cluster are shown above the bar graph. Error bars represent the SD. S1, mixed; S2, *M. catarrhalis/nonliquefaciens* dominated; S3, *H. aegyptius/influenzae* (OTU 3) dominated; S4, *S. pneumoniae* dominated; S5, *H. aegyptius/influenzae* (OTU 4) dominated; S6, *H. aegyptius/influenzae* (OTU 0) dominated.

Supplementary Figure 2: Rarefaction curves for bacterial OTU clustering at 99.6% sequence similarity in each sample.

(a) Including singletons (186 OTUs), (b) excluding singletons (69 OTUs), and (C) Good's coverage of each sample including and excluding singletons.

Supplementary Figure 3: Relative abundance of all genera in each sample.

The samples are ordered by clusters based on genus-level abundance for the seven most common genera (see Figure 2). G1, mixed; G2, *Moraxella* and *Streptococcus* dominated; G3, *Moraxella* dominated; G4, *Streptococcus* dominated; G5, *Haemophilus* and *Moraxella* dominated; G6, *Haemophilus* dominated.

Supplementary Figure 4: Relative abundance of all OTUs in each sample.

The samples are ordered by clusters based on species-level analysis (see Figure 3). If multiple species had the same RDP-Seqmatch score, the names of the species are separated with "/", for example, "*Moraxella catarrhalis/nonliquefaciens*". S1, mixed; S2, *M. catarrhalis/nonliquefaciens* dominated; S3, *H. aegyptius/influenzae* (OTU 3) dominated; S4, *S. pneumoniae* dominated; S5, *H. aegyptius/influenzae* (OTU 4) dominated; S6, *H. aegyptius/influenzae* (OTU 0) dominated.

Supplementary Figure 5: Results of the nested PCR for detection of the IOLA 16S rRNA gene.

The nested PCR products obtained using the IOLA-specific primer set were subjected to 2% agarose gel electrophoresis. Positive control, lane P; 100 bp DNA size marker, lane M.

Supplementary Figure 6: Phylogenetic analysis of the OTUs assigned as unclassified *Moraxella* species.

The 16S rRNA gene sequences assigned as *Moraxella* species by the RDP-Seqmatch program in this study were aligned with type strains of the genus *Moraxella* by using MAFFT. The maximum likelihood tree based on the alignments (556 bp) was constructed with IQ-TREE software. The TIM3+F+I+G4 model selected by Model Finder was used. The accession numbers are given following the taxonomic name. OTUs obtained in this study are shown in bold. *Acinetobacter radioresistens* X81666 was used as an outgroup. The scale bar denotes substitutions per site. Bootstrap values from 1,000 analyses are shown at the branch points.

Supplementary Figure 7: Phylogenetic analysis of the OTU assigned as unclassified *Helcococcus* species

The 16S rRNA gene sequence assigned as unclassified *Helcococcus* by the RDP-Seqmatch program in this study was aligned with type strains of the family Clostridiales Incertae Sedis XI by using MAFFT. The maximum likelihood tree based on the alignments (531 bp) was constructed with IQ-TREE software. The TVMe+R3 model selected by Model Finder was used. The accession numbers are given following the taxonomic name. OTUs obtained in this study are shown in bold. *Ruminococcus albus* L76598 was used as an outgroup. The scale bar denotes substitutions per site. Bootstrap values from 1,000 analyses are shown at the branch points.

Supplementary Figure 8: Phylogenetic analysis of the OTU assigned as unclassified *Flavobacterium* species.

The 16S rRNA gene sequence assigned as unclassified *Flavobacterium* by the RDP-Seqmatch program in this study was aligned with type strains of the genus *Flavobacterium* by using MAFFT. The maximum likelihood tree based on the alignments (561 bp) was constructed with IQ-TREE software. The TIM3e+I+G4 model selected by Model Finder was used. The accession numbers are given following the taxonomic name. OTUs obtained in this study are shown in bold. *Polaribacter franzmannii* U14586 was used as an outgroup. The scale bar denotes substitutions per site. Bootstrap values from 1,000 analyses are shown at the branch points.

Supplementary Figure 9: Phylogenetic analysis of the OTU assigned as unclassified *Rheinheimera* species.

The 16S rRNA gene sequence assigned as unclassified *Rheinheimera* by the RDP-Seqmatch program in this study was aligned with type strains of the genus *Rheinheimera* by using MAFFT. The maximum likelihood tree based on the alignments (552 bp) was constructed with IQ-TREE software. The TIM3e+I+G4 model selected by Model Finder was used. The accession numbers are given following the taxonomic name. OTUs obtained in this study are shown in bold. *Thiocapsa rosea* AJ006062 was used as an outgroup. The scale bar denotes substitutions per site. Bootstrap values from 1,000 analyses are shown at the branch points.

Supplementary Figure 10: Phylogenetic analysis of the OTU assigned as unclassified *Neisseriaceae* bacteria.

The 16S rRNA gene sequences assigned as unclassified *Neisseriaceae* bacteria by the RDP-Classifier program in this study were aligned with type strains of the order *Neisseriales* by using MAFFT. The maximum likelihood tree based on the alignments (556 bp) was constructed with IQ-TREE software. The TN+F+I+G4 model selected by Model Finder was used. The accession numbers are given following the taxonomic name. OTUs obtained in this study are shown in bold. *Burkholderia cepacia* U96927 was used as an outgroup. The scale bar denotes substitutions per site. Bootstrap values from 1,000 analyses are shown at the branch points.

Supplemental Figure 11: Phylogenetic analysis of the OTUs assigned as unclassified *Haemophilus* species.

The 16S rRNA gene sequences assigned as *Haemophilus* species by the RDP-Seqmatch program in this study were aligned with type strains of the genus *Haemophilus* by using MAFFT. The maximum likelihood tree based on the alignments (551 bp) was constructed with IQ-TREE software. The TPM3u+F+I+G4 model selected by Model Finder was used. The accession numbers are given following the taxonomic name. OTUs obtained in this study are shown in bold. *Aeromonas salmonicida* AJ009859 was used as an outgroup. The scale bar denotes substitutions per site. Bootstrap values from 1,000 analyses are shown at the branch points.

Supplemental Figure 12: Phylogenetic analysis of the OTU assigned as unclassified *Pseudomonas* species.

The 16S rRNA gene sequence assigned as unclassified *Pseudomonas* by the RDP-Seqmatch program in this study was aligned with type strains of the genus *Pseudomonas* by using MAFFT. The maximum likelihood tree based on the alignments (549 bp) was constructed with IQ-TREE software. The K2P+R6 model selected by Model Finder was used. The accession numbers are given following the taxonomic name. OTUs obtained in this study are shown in bold. *Cellvibrio vulgaris AF448513* was used as an outgroup. The scale bar denotes substitutions per site. Bootstrap values from 1,000 analyses are shown at the branch points

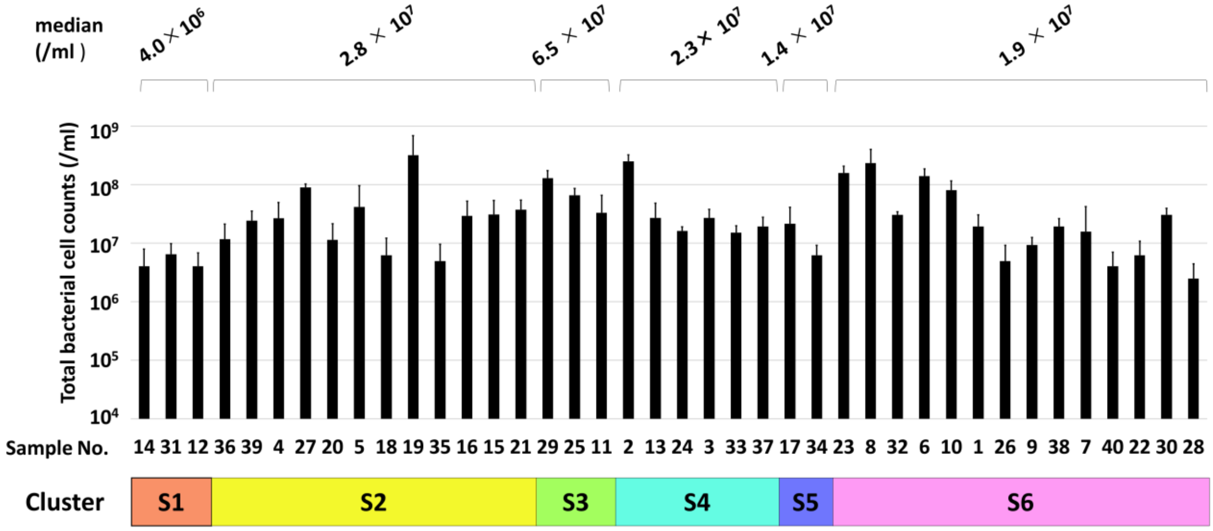

Supplementary Figure 1: Total bacterial cell counts in each sample.
The samples are ordered by clusters based on species-level analysis (see Figure 3). The medians of the total bacterial cell count per cluster are shown above the bar graph. Error bars represent the SD. S1, mixed; S2, *M. catarrhalis/nonliquefaciens* dominated; S3, *H. aegyptius/influenzae* (OTU 3) dominated; S4, *S. pneumoniae* dominated; S5, *H. aegyptius/influenzae* (OTU 4) dominated; S6, *H. aegyptius/influenzae* (OTU 0) dominated.

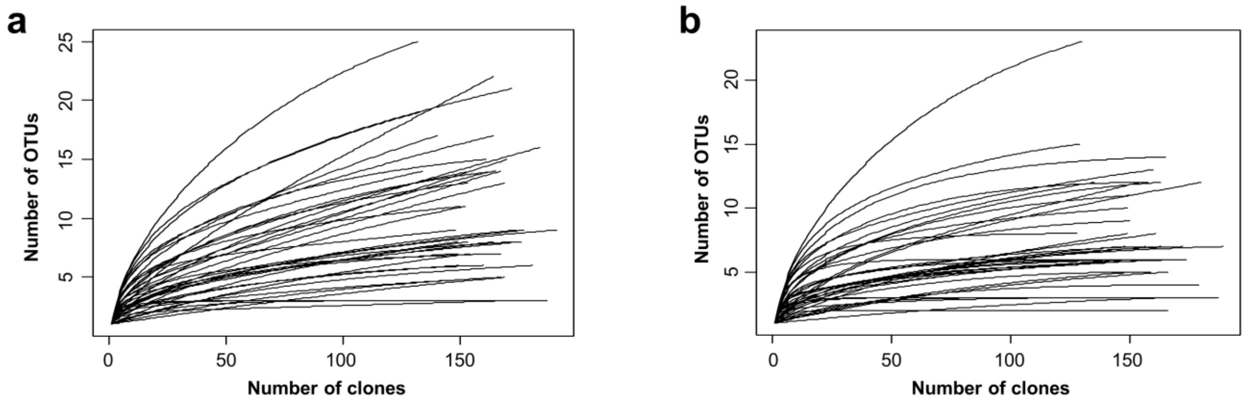

c

Sample No.		1	2	3	4	5	6	7	8	9	10	11	12	13	14	15	16	17	18	19	20
Good's coverage	Including singletons	0.94	0.94	0.90	0.96	0.99	0.94	0.95	0.96	0.99	0.99	0.98	0.93	0.95	0.95	0.99	0.97	0.99	0.97	0.99	0.98
	Excluding singletons	0.98	0.99	0.95	0.99	0.99	0.97	0.97	1.00	0.99	1.00	0.99	0.95	0.99	0.99	1.00	0.97	1.00	0.99	1.00	0.99

Sample No.		21	22	23	24	25	26	27	28	29	30	31	32	33	34	35	36	37	38	39	40
Good's coverage	Including singletons	0.99	1.00	0.98	0.95	1.00	0.98	0.98	0.99	0.96	0.98	0.95	0.98	0.97	1.00	0.99	0.95	0.98	0.97	0.98	0.98
	Excluding singletons	0.99	1.00	1.00	0.99	1.00	0.99	0.99	1.00	0.99	0.99	0.98	0.99	0.99	1.00	1.00	0.98	0.99	0.99	1.00	0.99

Supplementary Figure 2: Rarefaction curves for bacterial OTU clustering at 99.6% sequence similarity in each sample.

(a) Including singletons (186 OTUs), (b) excluding singletons (69 OTUs), and (C) Good's coverage of each sample including and excluding singletons.

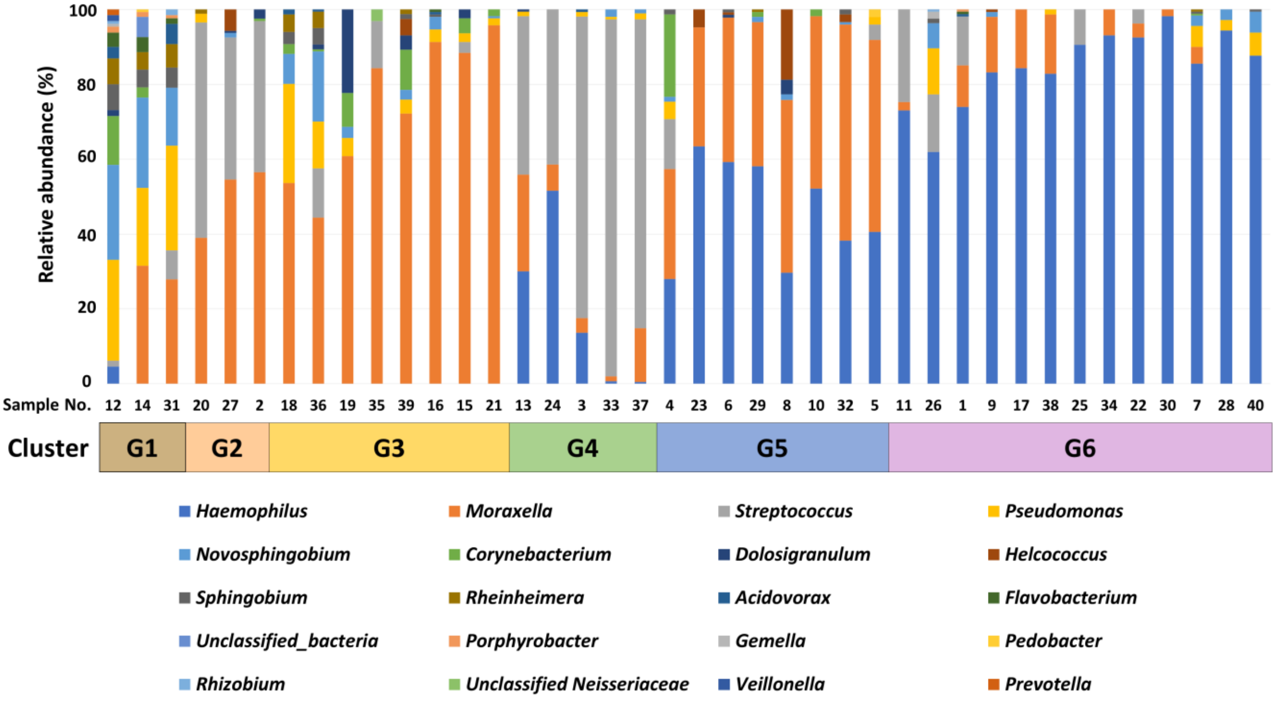

Supplementary Figure 3: Relative abundance of all genera in each sample.
The samples are ordered by clusters based on genus-level abundance for the seven most common genera (see Figure 2). G1, mixed; G2, *Moraxella* and *Streptococcus* dominated; G3, *Moraxella* dominated; G4, *Streptococcus* dominated; G5, *Haemophilus* and *Moraxella* dominated; G6, *Haemophilus* dominated.

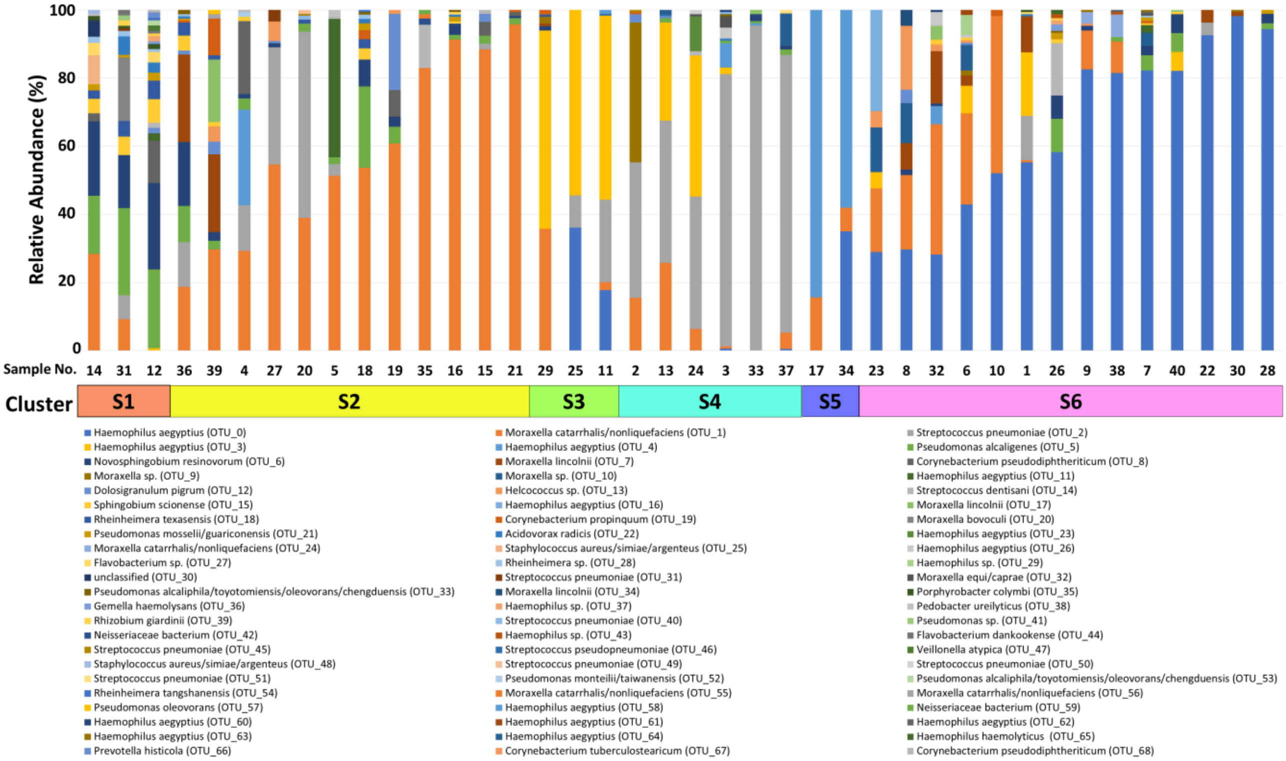

Supplementary Figure 4: Relative abundance of all OTUs in each sample.

The samples are ordered by clusters based on species-level analysis (see Figure 3). If multiple species had the same RDP-Seqmatch score, the names of the species are separated with "/", for example, "*Moraxella catarrhalis/nonliquefaciens*". S1, mixed; S2, *M. catarrhalis/nonliquefaciens* dominated; S3, *H. aegyptius/influenzae* (OTU 3) dominated; S4, *S. pneumoniae* dominated; S5, *H. aegyptius/influenzae* (OTU 4) dominated; S6, *H. aegyptius/influenzae* (OTU 0) dominated.

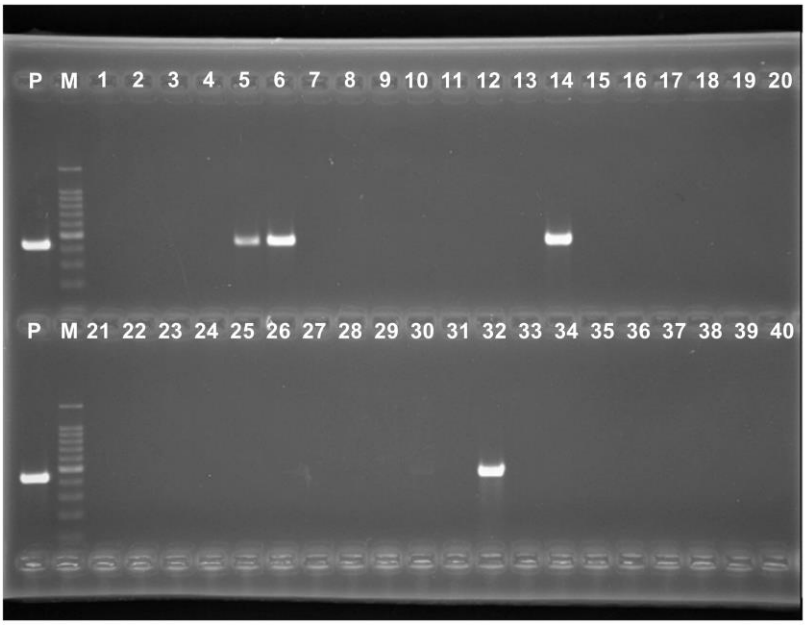

Supplementary Figure 5: Results of the nested PCR for detection of the IOLA 16S rRNA gene.

The nested PCR products obtained using the IOLA-specific primer set were subjected to 2% agarose gel electrophoresis. Positive control, lane P; 100 bp DNA size marker, lane M.

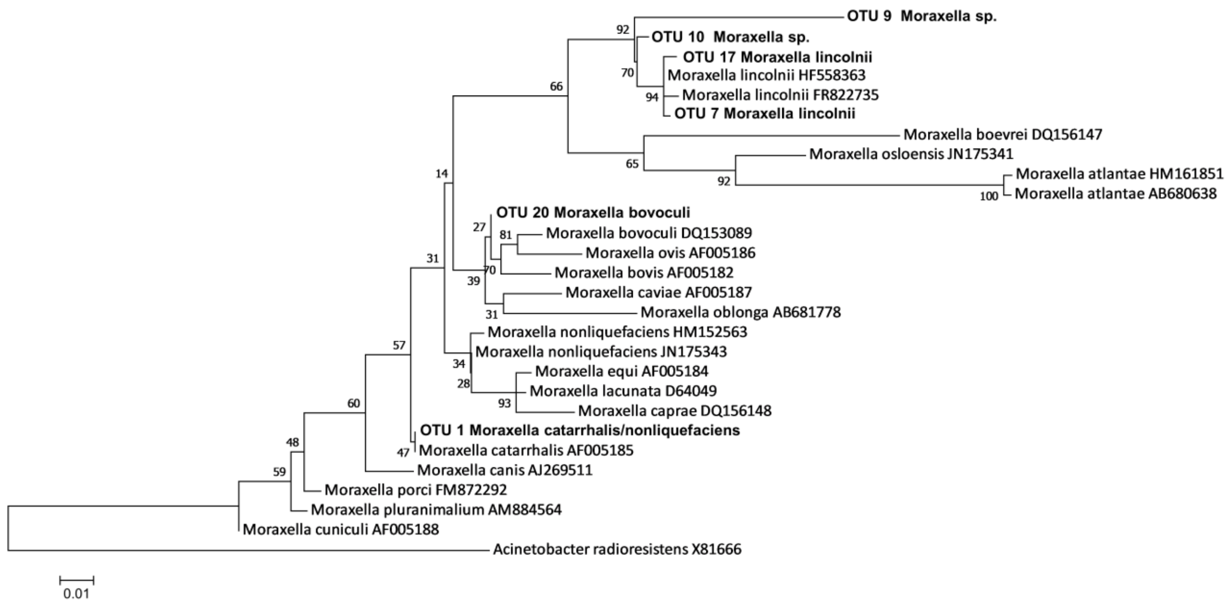

Supplementary Figure 6: Phylogenetic analysis of the OTUs assigned as unclassified *Moraxella* species.

The 16S rRNA gene sequences assigned as *Moraxella* species by the RDP-Seqmatch program in this study were aligned with type strains of the genus *Moraxella* by using MAFFT. The maximum likelihood tree based on the alignments (556 bp) was constructed with IQ-TREE software. The TIM3+F+I+G4 model selected by Model Finder was used. The accession numbers are given following the taxonomic name. OTUs obtained in this study are shown in bold. *Acinetobacter radioresistens* X81666 was used as an outgroup. The scale bar denotes substitutions per site. Bootstrap values from 1,000 analyses are shown at the branch points.

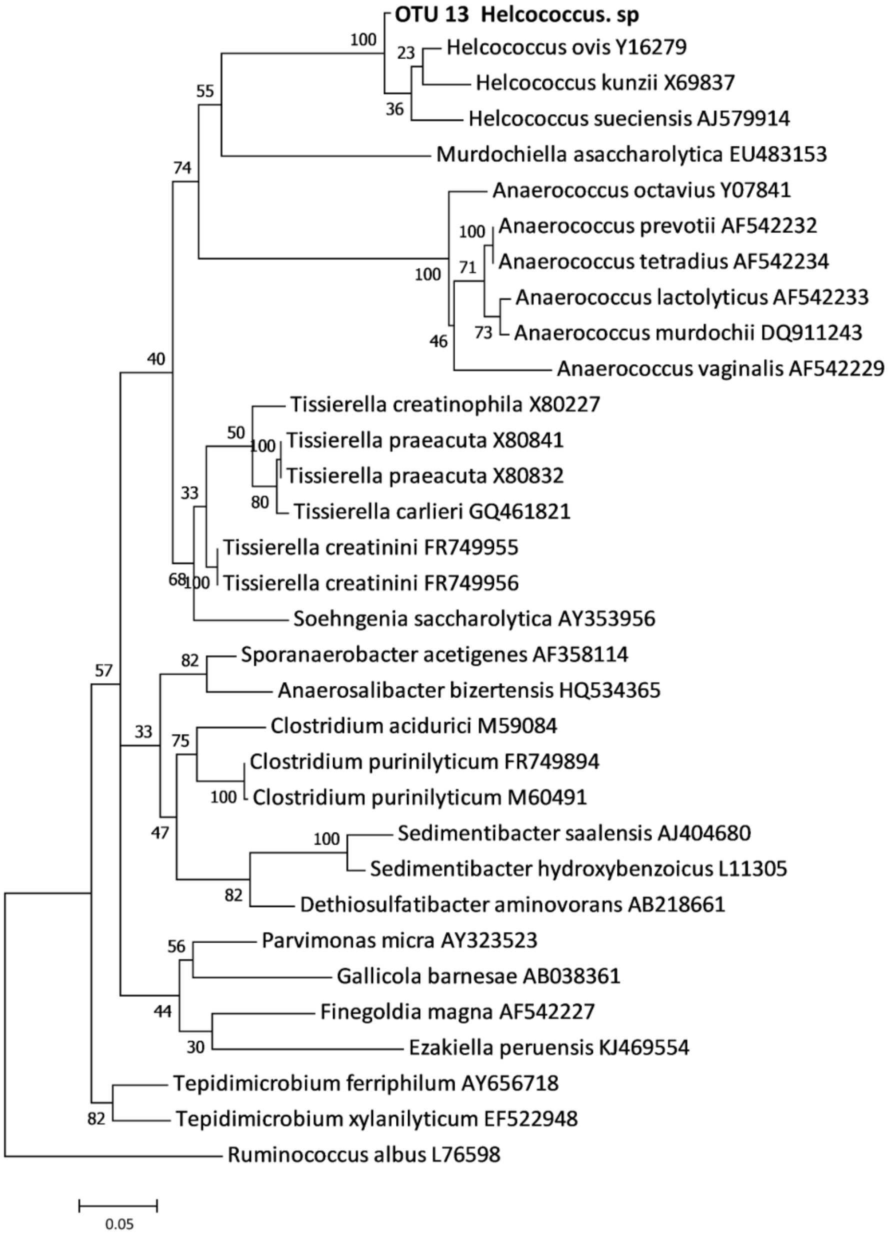

Supplementary Figure 7: Phylogenetic analysis of the OTU assigned as unclassified *Helcococcus* species

The 16S rRNA gene sequence assigned as unclassified *Helcococcus* by the RDP-Seqmatch program in this study was aligned with type strains of the family Clostridiales Incertae Sedis XI by using MAFFT. The maximum likelihood tree based on the alignments (531 bp) was constructed with IQ-TREE software. The TVMe+R3 model selected by Model Finder was used. The accession numbers are given following the taxonomic name. OTUs obtained in this study are shown in bold. *Ruminococcus albus* L76598 was used as an outgroup. The scale bar denotes substitutions per site. Bootstrap values from 1,000 analyses are shown at the branch points.

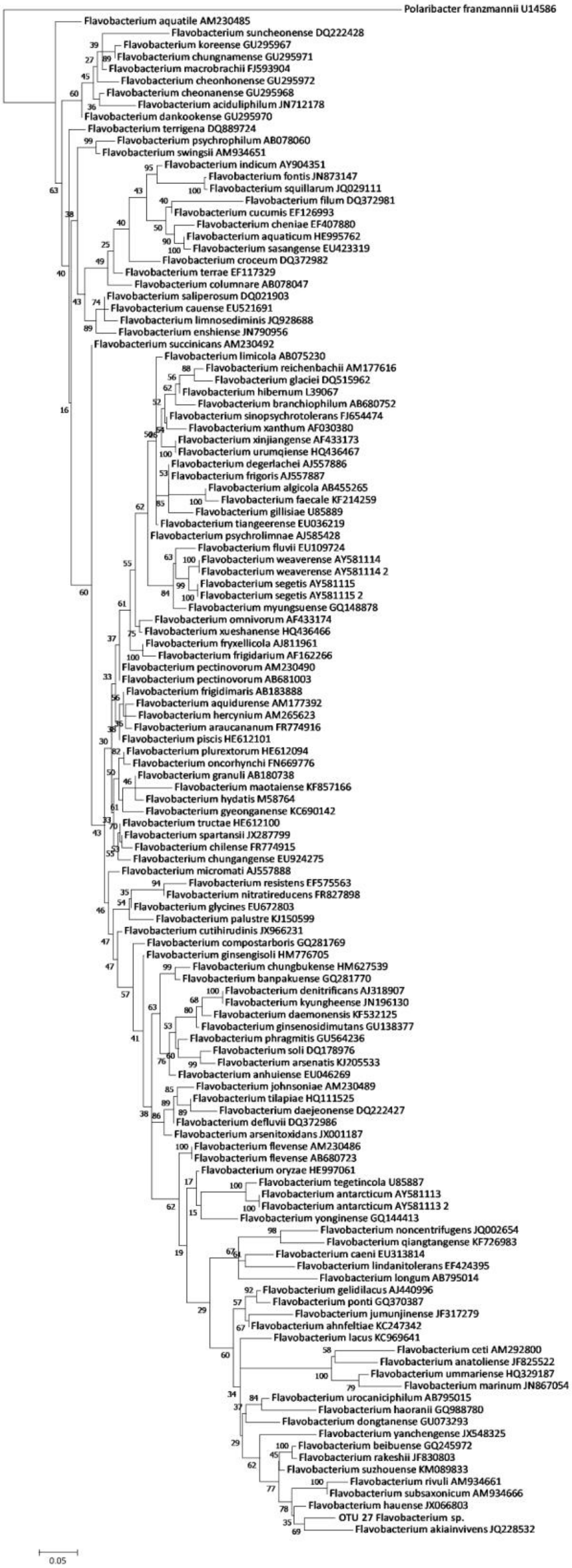

Supplementary Figure 8: Phylogenetic analysis of the OTU assigned as unclassified *Flavobacterium* species.

The 16S rRNA gene sequence assigned as unclassified *Flavobacterium* by the RDP-Seqmatch program in this study was aligned with type strains of the genus *Flavobacterium* by using MAFFT. The maximum likelihood tree based on the alignments (561 bp) was constructed with IQ-TREE software. The TIM3e+I+G4 model selected by Model Finder was used. The accession numbers are given following the taxonomic name. OTUs obtained in this study are shown in bold. *Polaribacter franzmannii* U14586 was used as an outgroup. The scale bar denotes substitutions per site. Bootstrap values from 1,000 analyses are shown at the branch points.

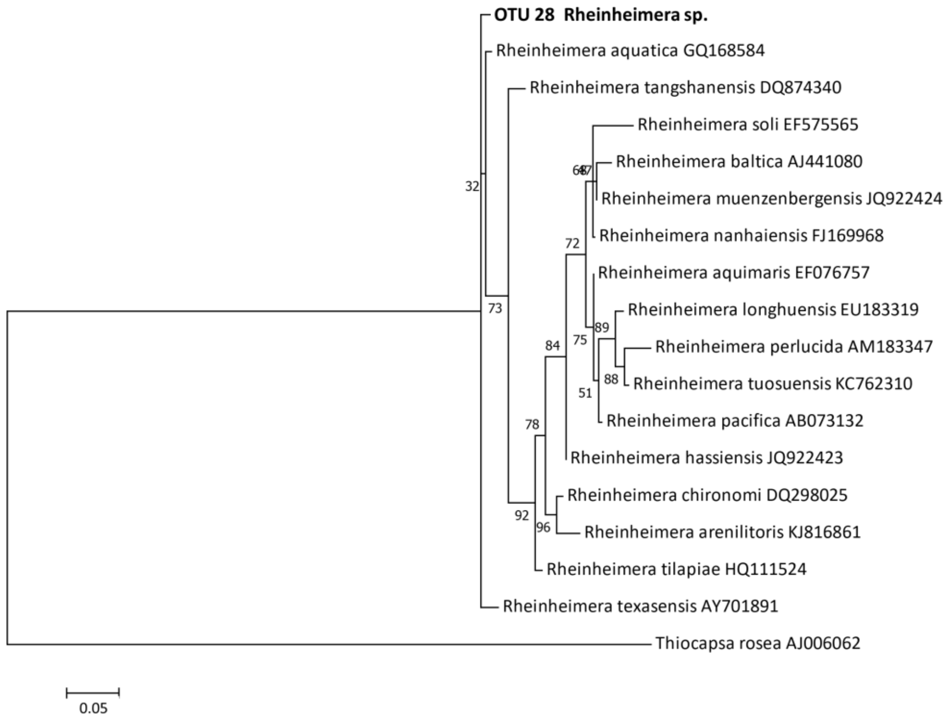

Supplementary Figure 9: Phylogenetic analysis of the OTU assigned as unclassified *Rheinheimera* species.

The 16S rRNA gene sequence assigned as unclassified *Rheinheimera* by the RDP-Seqmatch program in this study was aligned with type strains of the genus *Rheinheimera* by using MAFFT. The maximum likelihood tree based on the alignments (552 bp) was constructed with IQ-TREE software. The TIM3e+I+G4 model selected by Model Finder was used. The accession numbers are given following the taxonomic name. OTUs obtained in this study are shown in bold. *Thiocapsa rosea* AJ006062 was used as an outgroup. The scale bar denotes substitutions per site. Bootstrap values from 1,000 analyses are shown at the branch points.

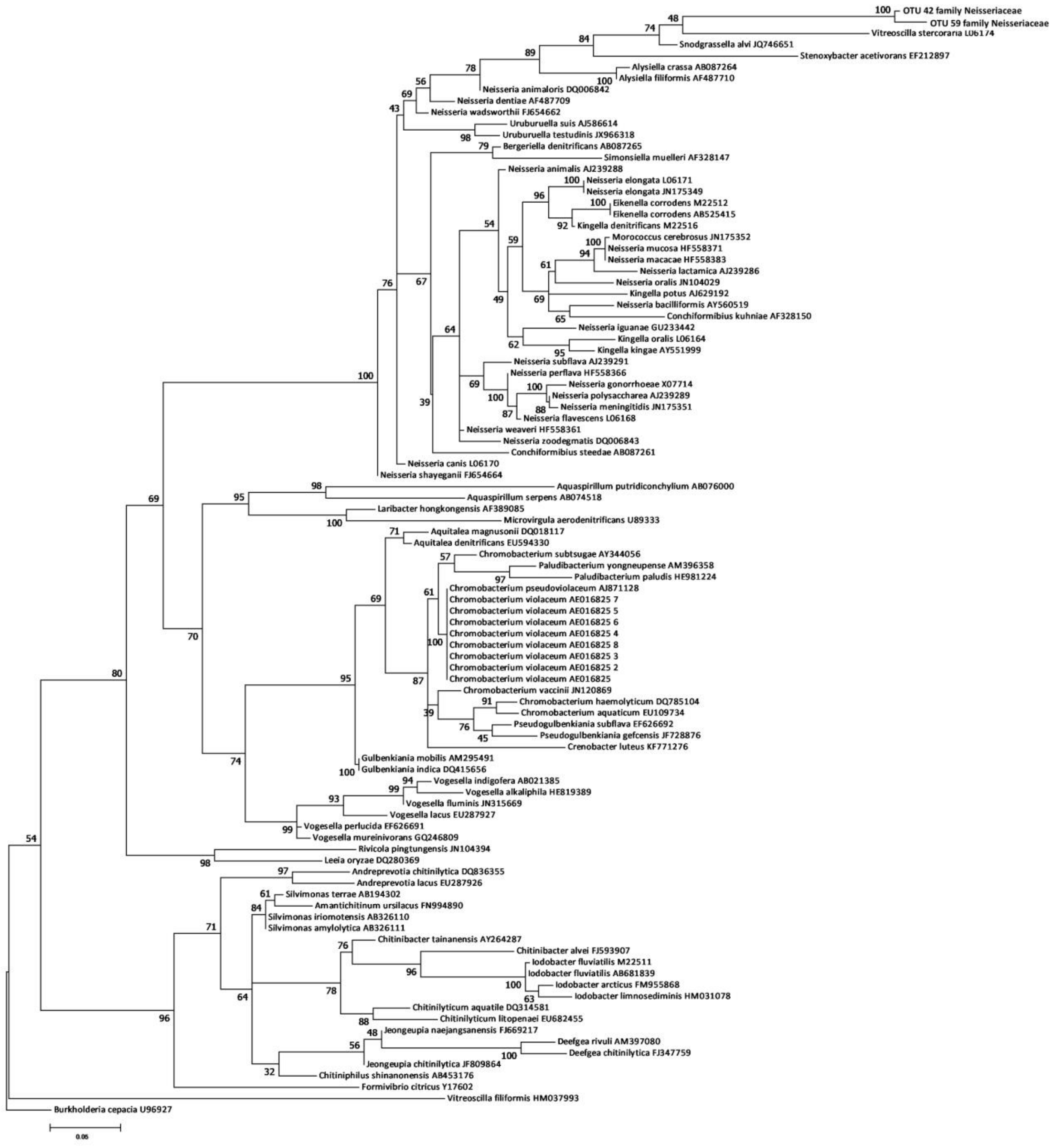

Supplementary Figure 10: Phylogenetic analysis of the OTU assigned as unclassified *Neisseriaceae* bacteria.

The 16S rRNA gene sequences assigned as unclassified *Neisseriaceae* bacteria by the RDP-Classifier program in this study were aligned with type strains of the order *Neisseriales* by using MAFFT. The maximum likelihood tree based on the alignments (556 bp) was constructed with IQ-TREE software. The TN+F+I+G4 model selected by Model Finder was used. The accession numbers are given following the taxonomic name. OTUs obtained in this study are shown in bold. *Burkholderia cepacia* U96927 was used as an outgroup. The scale bar denotes substitutions per site. Bootstrap values from 1,000 analyses are shown at the branch points.

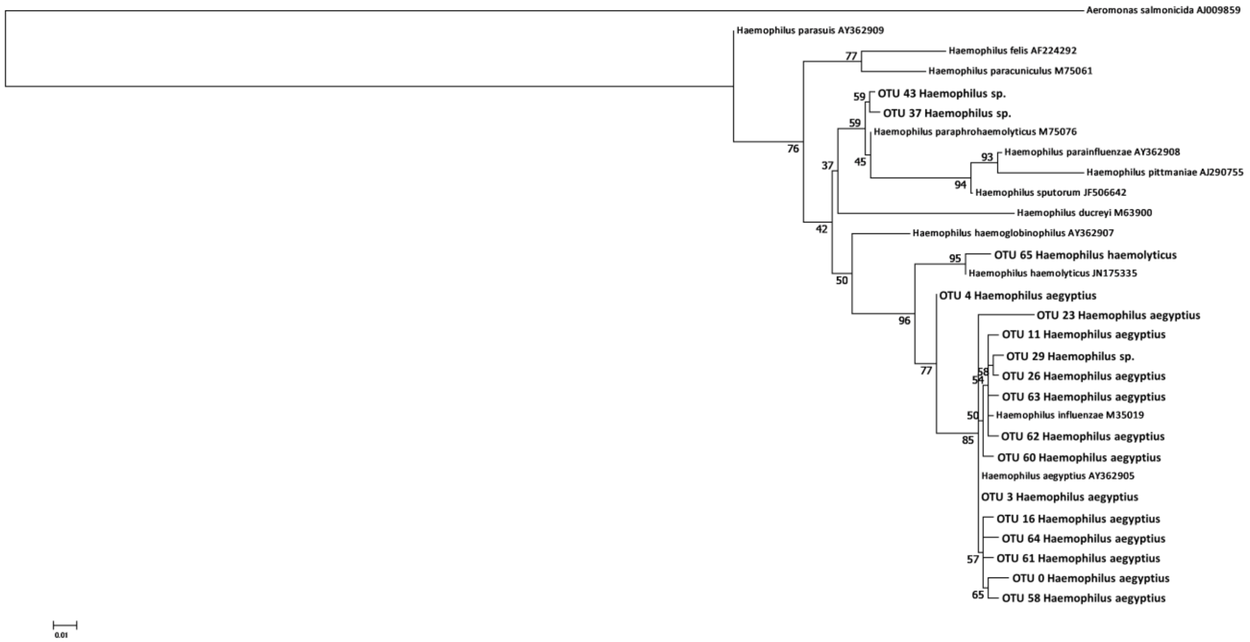

Supplemental Figure 11: Phylogenetic analysis of the OTUs assigned as unclassified *Haemophilus* species.

The 16S rRNA gene sequences assigned as *Haemophilus* species by the RDP-Seqmatch program in this study were aligned with type strains of the genus *Haemophilus* by using MAFFT. The maximum likelihood tree based on the alignments (551 bp) was constructed with IQ-TREE software. The TPM3u+F+I+G4 model selected by Model Finder was used. The accession numbers are given following the taxonomic name. OTUs obtained in this study are shown in bold. *Aeromonas salmonicida* AJ009859 was used as an outgroup. The scale bar denotes substitutions per site. Bootstrap values from 1,000 analyses are shown at the branch points.

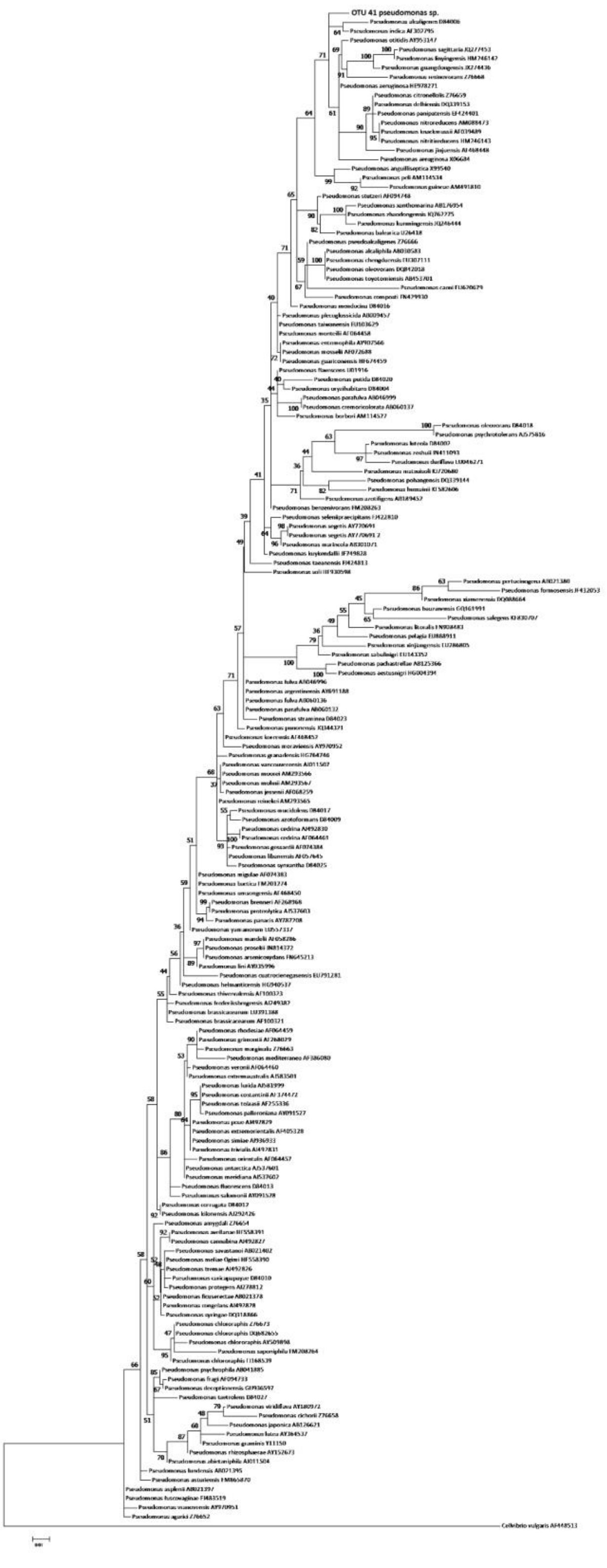

Supplemental Figure 12: Phylogenetic analysis of the OTU assigned as unclassified *Pseudomonas* species.

The 16S rRNA gene sequence assigned as unclassified *Pseudomonas* by the RDP-Seqmatch program in this study was aligned with type strains of the genus *Pseudomonas* by using MAFFT. The maximum likelihood tree based on the alignments (549 bp) was constructed with IQ-TREE software. The K2P+R6 model selected by Model Finder was used. The accession numbers are given following the taxonomic name. OTUs obtained in this study are shown in bold. *Cellvibrio vulgaris AF448513* was used as an outgroup. The scale bar denotes substitutions per site. Bootstrap values from 1,000 analyses are shown at the branch points.
